# Mecp2 regulates *tnfa* during zebrafish embryonic development and acute inflammation

**DOI:** 10.1242/dmm.026922

**Published:** 2017-12-01

**Authors:** M. van der Vaart, O. Svoboda, B. G. Weijts, R. Espín-Palazón, V. Sapp, T. Pietri, M. Bagnat, A. R. Muotri, D. Traver

**Affiliations:** 1 Department of Cellular and Molecular Medicine, University of California at San Diego, La Jolla, 92093 CA, USA; 2 Federated Department of Biological Sciences, New Jersey Institute of Technology, Newark, 07102 NJ, USA; 3 Department of Cell Biology, Duke University, Durham, 27708 NC, USA; 4 Department of Pediatrics/Rady Children's Hospital San Diego, School of Medicine, University of California San Diego, La Jolla, 92093 CA, USA; 5 Section of Cell and Developmental Biology, Division of Biological Sciences, University of California San Diego, La Jolla, 92093 CA, USA

**Keywords:** Inflammation, *mecp2*, *tnfa*, Zebrafish, Rett syndrome

## Abstract

Mutations in *MECP2* cause Rett syndrome, a severe neurological disorder with autism-like features. Duplication of *MECP2* also causes severe neuropathology. Both diseases display immunological abnormalities that suggest a role for MECP2 in controlling immune and inflammatory responses. Here, we used *mecp2*-null zebrafish to study the potential function of Mecp2 as an immunological regulator. Mecp2 deficiency resulted in an increase in neutrophil infiltration and upregulated expression of the pro- and anti-inflammatory cytokines Il1b and Il10 as a secondary response to disturbances in tissue homeostasis. By contrast, expression of the proinflammatory cytokine tumor necrosis factor alpha (Tnfa) was consistently downregulated in *mecp2*-null animals during development, representing the earliest developmental phenotype described for MECP2 deficiency to date. Expression of *tnfa* was unresponsive to inflammatory stimulation, and was partially restored by re-expression of functional *mecp2*. Thus, Mecp2 is required for *tnfa* expression during zebrafish development and inflammation. Finally, RNA sequencing of *mecp2*-null embryos revealed dysregulated processes predictive for Rett syndrome phenotypes.

## Abstract (precis)

**Summary:** As shown by evaluating the levels of pro- and anti-inflammatory cytokines in *mecp2*-null zebrafish, Mecp2 is required for *tnfa* expression during zebrafish development and inflammation.

## INTRODUCTION

The human X-chromosomal gene methyl-CpG-binding protein 2 (*MECP2*) was identified as an epigenetic factor capable of binding to methylated DNA (Lewis et al., 1992). Mutations in human *MECP2* lead to Rett syndrome (RTT) (Amir et al., 1999), a severe neurological disorder associated with autistic features and motor skill regression after an apparently normal early development (Lyst and Bird, 2015). RTT patients often also display growth retardation (Tarquinio et al., 2012), gastrointestinal (GI) and biliary tract disorders (Motil et al., 2012) and oxidative stress (Filosa et al., 2015). Conversely, overexpression of human *MECP2* caused by duplication of its genetic locus (Xq28) results in severe mental retardation and progressive neurological symptoms (Van Esch et al., 2005). Although neurological defects are the most striking clinical presentation of RTT and MECP2-duplication syndrome, both diseases display immunological abnormalities that point towards a role for MECP2 in regulating immune and inflammatory responses.

Disturbances in tissue homeostasis are detected by pattern recognition receptors (PRRs), such as the family of Toll-like receptors (TLRs) that recognize pathogen-associated molecular patterns (PAMPs) or damage-associated molecular patterns (DAMPs) (Beg, 2002; Matzinger, 2002; Medzhitov and Janeway, 2000). Activation of TLRs by infection or cellular damage initiates a signaling cascade that leads to the production of proinflammatory cytokines and chemokines (Akira and Takeda, 2004). The primary function of proinflammatory cytokines, including tumor necrosis factor alpha (TNFα) and interleukin 1 beta (IL1B), is to initiate an appropriate cellular or humoral immune response to neutralize the disturbance. Anti-inflammatory cytokines, including interleukin 10 (IL10) and transforming growth factor beta (TGFB), balance the activity of proinflammatory cytokines by stimulating resolution of inflammation and tissue repair. Alterations in the balance between pro- and anti-inflammatory cytokines are potentially harmful, as prolonged inflammation can be damaging to tissues, while inadequate immune responses leave the body vulnerable to infections.

RTT patients showed a dysregulated cytokine and chemokine profile and displayed subclinical inflammation (Cortelazzo et al., 2014; Pecorelli et al., 2016). Data obtained using a mouse model of RTT demonstrated that MECP2 regulates microglia and macrophage responsiveness to inflammatory stimulation, hypoxia and glucocorticoids (Cronk et al., 2015). Transplantation of wild-type microglia has even been suggested as a therapeutic strategy for RTT patients based on findings obtained using RTT mice (Derecki et al., 2012), but these findings have since been disputed by others in the field (Wang et al., 2015). Although investigations concerning the role of the immune system in the onset of RTT are ongoing, MECP2 duplication syndrome is linked to immunodeficiency with increased susceptibility to infections for reasons that remain to be uncovered (Bauer et al., 2015). An emerging theme is that MECP2 normally regulates the immune response towards inflammatory stimuli and other stress factors.

The zebrafish was originally employed as a model organism to study vertebrate embryogenesis because of its external fertilization and development, genetic tractability, and optical transparency allowing noninvasive intravital imaging (Kimmel et al., 1988). These characteristics have also helped to develop the zebrafish as a useful model for the study of vertebrate immunity (Renshaw and Trede, 2011; van der Vaart et al., 2012). A recently described *mecp2*-null zebrafish mutant showed altered motor behaviors (Pietri et al., 2013), and *mecp2* was found to be required for normal zebrafish brain development (Gao et al., 2015). Zebrafish *mecp2* was broadly expressed early in embryonic development, after which it became enriched in the brains of zebrafish larvae (Gao et al., 2015). This is similar to the distribution of MECP2 in mice, where it is highly expressed in neurons, but also ubiquitously found at lower levels in other cell types (Song et al., 2014).

Here, we studied the potential function of zebrafish Mecp2 as an immunological regulator during development and inflammation. We found that *mecp2*-null zebrafish display several previously unappreciated phenotypes also present in RTT patients, including growth retardation, GI tract phenotypes and dysregulated expression of cytokines. The gene expression levels of the pro- and anti-inflammatory cytokines *il1b* and *il10* showed a peak during development, but were not hyper-responsive to inflammatory stimulation in *mecp2*-null larvae. We therefore suggest that the increased expression levels of these inflammatory cytokines during development were a response to a disruption of tissue homeostasis in the absence of Mecp2. Remarkably, we found that the expression levels of zebrafish *tnfa* were profoundly downregulated during the first hours of development in *mecp2*-null embryos, preceding the first noticeable disease phenotypes. To the best of our knowledge, this finding represents the earliest developmental phenotype associated with MECP2-deficieny. The lower *tnfa* expression levels persisted throughout larval development, and *tnfa* was unresponsive to inflammatory stimulation in *mecp2*-null larvae. Finally, the expression of *tnfa* in *mecp2*-null embryos could be partially restored by enforced expression of wild-type *mecp2*. However, re-expression of *tnfa* in *mecp2*-null embryos was not sufficient to rescue the observed RTT phenotypes. Based on these findings, we conclude that zebrafish Mecp2 is required for *tnfa* expression during development and inflammation. To assess the earliest changes attributable to loss of Mecp2 function, we utilized RNA sequencing to analyze the transcriptome of *mecp2*-null embryos shortly after initiation of embryonic transcription (Kane and Kimmel, 1993). Strikingly, this revealed disrupted biological processes that are highly predictive of RTT phenotypes that develop much later in human patients. Further exploration of this transcriptome data and its changes over time might generate novel insights into additional developmental functions of MECP2.

## RESULTS

### *mecp2*-null zebrafish display growth retardation, GI tract phenotypes and systemic inflammation

**Fig. 1.**
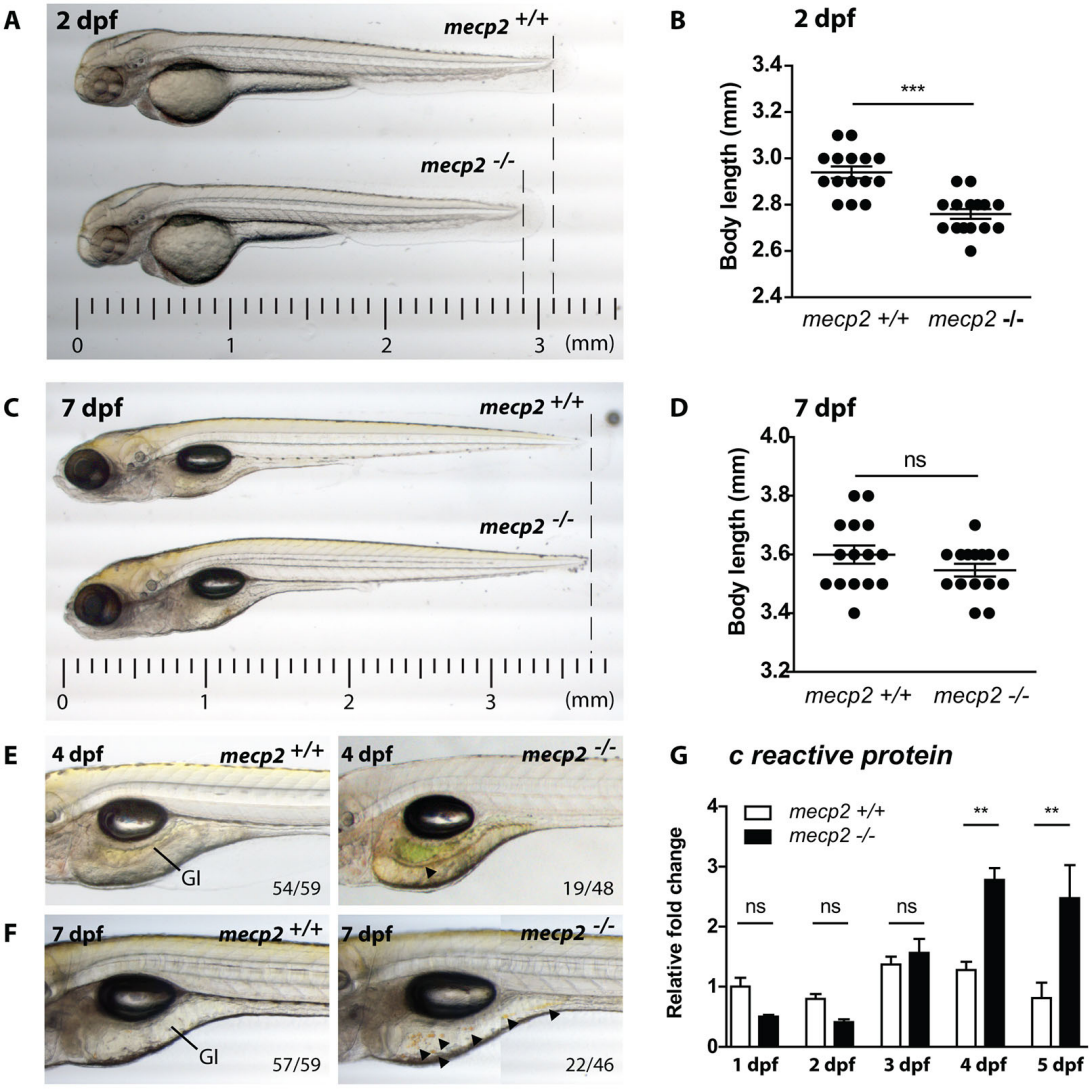
***mecp2*-deficient zebrafish display inflammation during larval development.** (A,C) Representative stereo microscopy images of 2 dpf and 7 dpf wild-type and *mecp2*-null zebrafish larvae. (B,D) Total body lengths of the 2 dpf and 7 dpf wild-type and *mecp2*-null larvae, as measured in millimeters (*n*=15 per condition; Student’s *t*-test; ***P<0.001; data are representative of three individual experiments). (E,F) Stereo microscopy images of 4 dpf and 7 dpf wild-type and *mecp2*-null zebrafish illustrating the GI tract phenotypes regularly observed (indicated by arrowheads). The frequency of these phenotypes is shown in relation to the total number of examined animals (19 of 48 *mecp2*-null animals at 3 dpf; 22 of 46 *mecp2*-null animals at 7 dpf). (G) qPCR was performed to determine the whole-organism gene expression level of the inflammation marker *crp* relative to the expression of the housekeeping gene *tbp*. Wild-type and *mecp2*-null samples (*n*=3 with 20 embryos or larvae pooled per sample) were taken every day for the first 5 days of development. The relative fold change versus gene expression in a 1 dpf wild type is shown (one-way ANOVA with Tukey's post hoc test; **P<0.01; ns, not significant; data are representative of two individual experiments).

To study the function of Mecp2 during zebrafish development, we used a mutant line containing a premature stop codon in the *mecp2* gene (mecp2^Q63*^) that truncates the protein before the methyl binding domain (MBD) and transcriptional repression domain (TRD), both of which are vital to its function (Lyst and Bird, 2015; Pietri et al., 2013). Although adult *mecp2*-null zebrafish are viable and fertile with no overt phenotypes, these animals display behavioral alterations during their larval development (Pietri et al., 2013). Upon further characterization, we found that developing *mecp2*-null embryos displayed growth retardation at 2 days postfertilization (dpf) (Fig. 1A,B). However, no significant difference in total body length was discernible between *mecp2*-null and wild-type embryos at 7 dpf (Fig. 1C,D). At ∼4 dpf, green/yellow discoloration was observed in the GI tracts of *mecp2*-null larvae (Fig. 1E), indicative of an accumulation of, or disruption in, flow of bile (Delous et al., 2012). At 7 dpf, dark yellow droplets were regularly observed in the GI tracts of *mecp2*-null larvae (Fig. 1F), consistent with bile overproduction. To investigate whether these phenotypes are preceded or accompanied by systemic inflammation, we analyzed gene expression of the inflammation marker C reactive protein (*crp*) by quantitative real-time PCR (qPCR) (Okamura et al., 1990). In the first 3 days of zebrafish development, we found no difference in *crp* expression between wild-type and *mecp2*-null larvae, but *crp* levels were significantly elevated in *mecp2*-null larvae by 4 dpf and 5 dpf (Fig. 1G). This demonstrates that *mecp2*-null larvae mount an inflammatory response at 4 dpf and 5 dpf that is detectable at a whole-organism level, after an early developmental period with no overt signs of systemic inflammation. Together, these results show that *mecp2*-null zebrafish display several RTT features during their development, including growth retardation, GI tract phenotypes and systemic inflammation.

### Neutrophil numbers and mobilization confirm the presence of inflammation in *mecp2*-null larvae

**Fig. 2.**
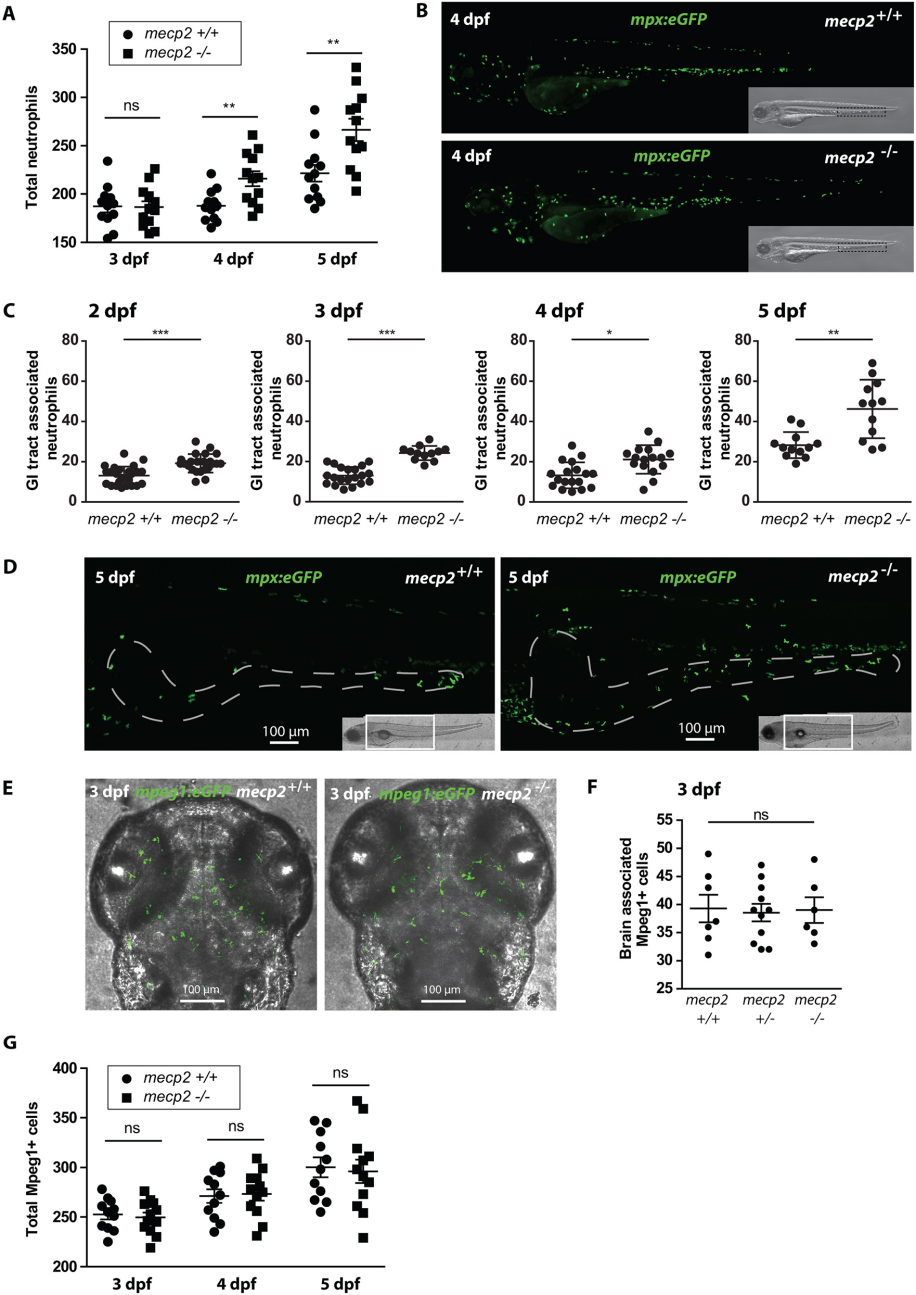
**Neutrophil number and distribution confirm the presence of inflammation in *mecp2*-null larvae.** (A) Total numbers of Tg(*mpx*:eGFP)-positive neutrophils were counted in 3, 4 and 5 dpf wild-type and *mecp2*-null larvae using stereo fluorescent microscopy (*n*=12 larvae per condition pooled from two individual experiments; larvae were scored for three consecutive days). (B) Representative stereo microscopy images of 4 dpf Tg(*mpx*:eGFP) wild-type and *mecp2*-null larvae. (C) Numbers of Tg(*mpx*:eGFP)-positive neutrophils associated with the GI tract of 2, 3, 4 and 5 dpf wild-type and *mecp2*-null larvae were counted (*n*≥12 embryos per condition; data are representative of three individual experiments). (D) Representative confocal micrographs (maximum projection) of the GI tracts of 5 dpf Tg(*mpx*:eGFP) wild-type and *mecp2*-null larvae in which the GI tract has been delineated with a white dashed line based on the transmitted light images. (E) Representative confocal micrographs (maximum projection) of the brain region of 3 dpf Tg(*mpeg1*:eGFP) wild-type and *mecp2*-null larvae. (F) Brain-associated Tg(*mpeg1*:eGFP)-positive cells were counted for 3 dpf wild-type, heterozygous and *mecp2*-null larvae (*n*=7, *n*=11, *n*=6, respectively; one-way ANOVA with Tukey's post hoc test; ns, not significant; data are representative of two individual experiments). (G) Total numbers of Tg(*mpeg1*:eGFP)-positive cells were counted in 3, 4 and 5 dpf wild-type and *mecp2*-null larvae using stereo fluorescent microscopy (*n*=11 and *n*=12 embryos per condition, respectively; data are representative of two individual experiments). A Student’s *t*-test was used for all statistical analyses, except for the data analyzed in F, by comparing wild-type and *mecp2*-null numbers per day (***P<0.001; **P<0.01; *P<0.05; ns, not significant).

To further investigate and characterize the possible inflammatory response in *mecp2*-null larvae suggested by increased *crp* levels, we first analyzed neutrophil numbers. Neutrophils are among the first innate immune cells that respond to disturbances in tissue homeostasis; increased tissue infiltration has previously been used to mark inflammation in zebrafish models of wounding, infection and inflammatory bowel disease (Brudal et al., 2014; Brugman et al., 2009; Oehlers et al., 2011; Renshaw et al., 2006). We used Tg(*mpx*:eGFP) animals (Renshaw et al., 2006), in which neutrophils are fluorescently labeled, to assess the number and distribution of neutrophils in the *mecp2*-null background over several developmental time points. Correlating with our *crp* results, we did not find any difference in neutrophil number between wild-type and *mecp2*-null larvae at 3 dpf, but total neutrophil numbers were significantly increased in *mecp2*-null larvae at 4 dpf and 5 dpf (Fig. 2A,B). These findings reproduce the neutrophilia observed in *Mecp2*-null mice displaying RTT phenotypes, and underscore the conserved function of Mecp2 in lower vertebrates (Cronk et al., 2015).

Neutrophilic granulocytes begin to accumulate in the caudal hematopoietic tissue (CHT) of developing zebrafish embryos following initiation of circulation at 26 hpf (Bertrand et al., 2007; Le Guyader et al., 2008; Stachura and Traver, 2011). A large number of neutrophils continue to reside in the CHT in uninflamed larvae, from which they can be mobilized to migrate towards inflamed tissues when needed (Yoo and Huttenlocher, 2011). We therefore aimed to approximate the source of inflammation in *mecp2*-null larvae by determining which tissues displayed increased neutrophil infiltration. Although the head region of *mecp2*-null larvae contained a slightly increased number of neutrophils at 5 dpf, we did not observe any significant infiltration of neutrophils into the brains of *mecp2*-null animals (Fig. S1A-C). Starting at 2 dpf, we observed increases in neutrophil numbers associated with the GI tract of *mecp2*-null larvae (Fig. 2C,D), indicating this tissue as a potential source of inflammation. We reproduced this observation by using a previously characterized anti-sense morpholino oligonucleotide approach designed to block initiation of zebrafish Mecp2 protein translation (Fig. S1D) (Gao et al., 2015). These findings are in agreement with our previous observation of GI tract phenotypes during *mecp2*-null larval development.

Because microglia and macrophages have previously been implicated in RTT-like etiology in mice, and become depleted with disease progression (Cronk et al., 2015), we also assessed their number and localization by using Tg(*mpeg1*:eGFP) animals with fluorescently labeled microglia and macrophages (Ellett et al., 2011). At 3 dpf, *mpeg1*-expressing microglia have colonized the brain and are capable of mounting a functional immune response (Herbomel et al., 2001; Svahn et al., 2013). However, we found no distinguishable difference in microglia or macrophage numbers or localization between *mecp2*-null and wild-type larvae from 3 to 5 dpf (Fig. 2E-G). In summary, our results indicate that disrupting Mecp2 function during zebrafish development leads to a systemic immune response that appears to originate from the GI tract, based on observed GI tract phenotypes and neutrophil influx into this tissue.

### Expression of central pro- and anti-inflammatory cytokines is dysregulated in *mecp2*-null larvae

**Fig. 3.**
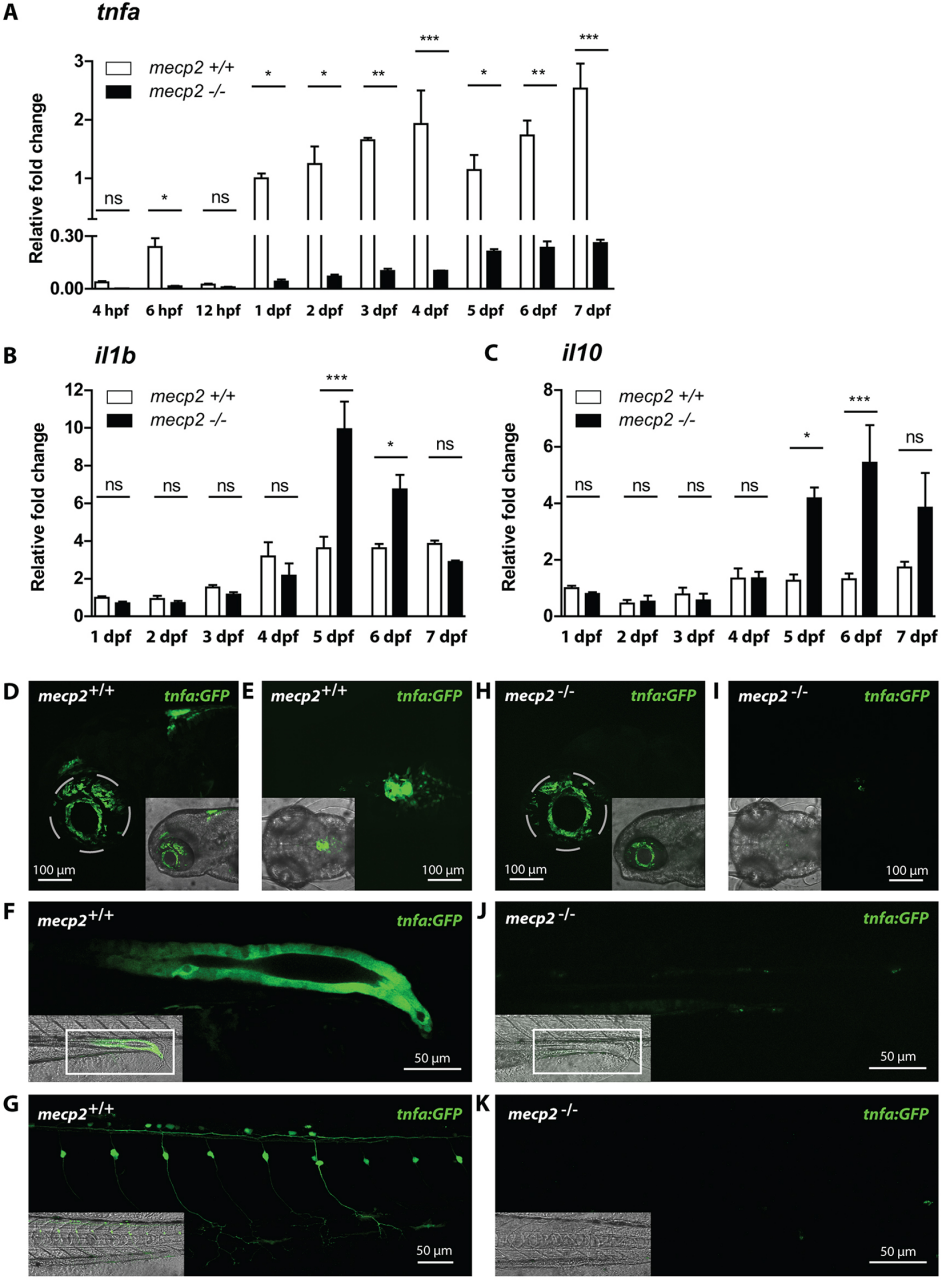
**Expression of central inflammatory cytokines is dysregulated in *mecp2*-null larvae.** (A,B,C) qPCR was performed to determine the whole-organism expression of *tnfa* from 4 hpf to 7 dpf (A), and *il1b* and *il10* from 1 dpf to 7 dpf (B,C), in wild-type or *mecp2*-null zebrafish. Gene expression is related to the expression of the housekeeping gene *tbp*, where the fold change relative to gene expression in 1 dpf wild-type embryos is shown (*n*=3 with 20 embryos or larvae pooled per sample for 1-7 dpf; 30 embryos were pooled per sample for the 4-12 hpf time points; data are representative of two individual experiments). One-way ANOVA with Tukey's post hoc test was used for all statistical analyses (***P<0.001; **P<0.01; *P<0.05; ns, not significant). (D-K) Representative confocal micrographs of 3 dpf Tg(*tnfa*:eGFP) wild-type and *mecp2*-null larvae showing the eGFP expression pattern in brain regions in a lateral view (D,H), brain regions in a dorsal view (E,I), posterior gut epithelium in a lateral view (F,J) and dorsal root ganglion neurons in a lateral view (G,K).

Because inflammation is mainly controlled by the expression and activity of pro- and anti-inflammatory cytokines and chemokines, we queried whether the expression of these regulatory molecules is affected by Mecp2 deficiency. We used qPCR to analyze the gene expression levels of a panel of zebrafish inflammatory cytokines and chemokines in *mecp2*-null and wild-type larvae during the first 7 days of development. The panel consisted of the proinflammatory cytokines *il1b*, interleukin 6 (*il6*) and *tnfa*; the proinflammatory chemokine interleukin 8 (*cxcl8a*); and the anti-inflammatory cytokines *il10* and *tgfb1*. At each time point analyzed over the 7-day time course, *tnfa* was expressed at dramatically lower levels in *mecp2*-null embryos and larvae compared with wild-type embryos and larvae of the same age (Fig. 3A). Even at 6 hpf, the earliest time point with clearly detectable *tnfa* expression in wild-type embryos, its expression was significantly reduced in *mecp2*-null animals (Fig. 3A). By contrast, we found no significant difference in expression levels of *il6*, *cxcl8a* and *tgfb1* between *mecp2*-null and wild-type animals over the developmental time course of 7 days (Fig. S2A-C). We did detect a significant increase in whole-organism *il1b* and *il10* expression in *mecp2*-null larvae at 5 dpf, after being expressed at wild-type levels for the first 4 days of development (Fig. 3B,C). While *il1b* reverted back to wild-type levels over the next 2 days (Fig. 3B), the significantly increased expression of *il10* peaked at 6 dpf, after which it also trended downwards (Fig. 3C). The expression levels of *il1b* and *il10* indicate a temporal increase in inflammatory signaling, followed by resolution of inflammation.

We sought to confirm the specific downregulation of *tnfa* by confocal microscopy imaging of wild-type and *mecp2*-null larvae carrying a Tg(*tnfa*:eGFP) reporter that expresses eGFP under control of *tnfa* regulatory sequences (Fig. S3) (Marjoram et al., 2015). Wild-type larvae of 3 dpf expressed eGFP in brain regions (Fig. 3D,E), posterior gut epithelium (Fig. 3F) and dorsal root ganglion neurons (Fig. 3G). By contrast, 3 dpf *mecp2*-null larvae had no detectable expression of GFP in any of these tissues (Fig. 3H-K). Because the Tg(*tnfa*:eGFP) reporter construct introduced an additional *tnfa* promoter region at a random location in the zebrafish genome (Marjoram et al., 2015), the lack of eGFP expression caused by Mecp2 deficiency appears to be linked to the DNA sequence of the *tnfa* promoter, rather than its chromosomal location. The decreased expression of *tnfa* precedes any observable phenotype, suggesting that this is not part of a secondary inflammatory response, but rather caused by genetic dysregulation.

### *mecp2*-null larvae are unable to activate *tnfa* expression during an acute inflammatory response

**Fig. 4.**
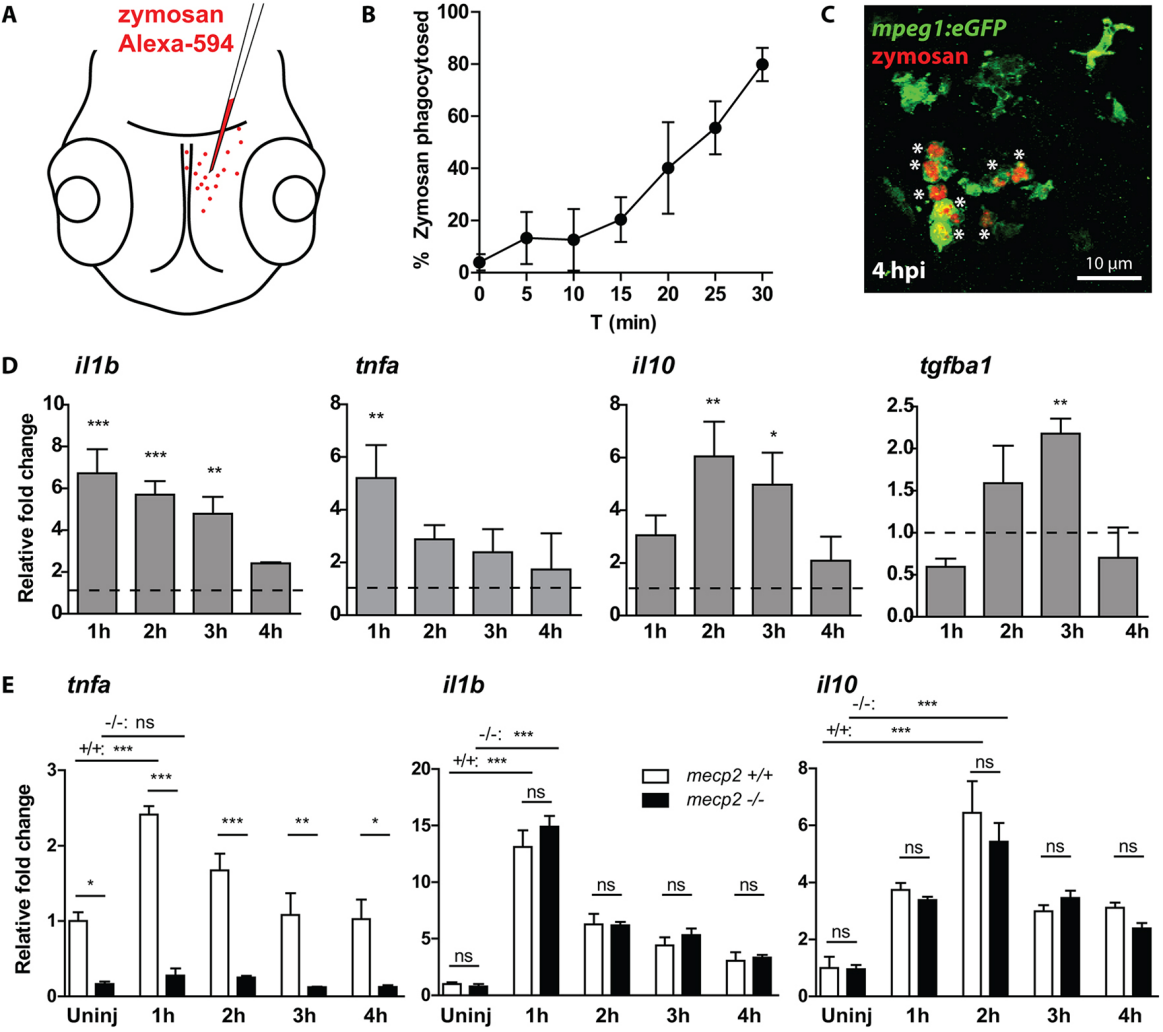
***mecp2*-null larvae are unable to increase *tnfa* expression during an acute inflammatory response.** (A) Schematic of the injection of Alexa Fluor 594-labeled zymosan into the brains of 3 dpf zebrafish larvae. (B) The percentage of zymosan particles phagocytosed by Tg(*mpeg1*:eGFP)-positive cells in wild-type larvae was determined using confocal microscopy of samples fixed every 5 min after injection (*n*=5 larvae per time point). (C) Representative confocal micrograph of a wild-type Tg(*mpeg1*:eGFP) larva at 4 hpi. Asterisks indicate zymosan particles phagocytosed by Tg(*mpeg1*:eGFP)-positive cells. (D) qPCR was performed to determine the whole-organism gene expression level of *il1b*, *tnfa*, *il10* and *tgfb1* relative to the expression of the housekeeping gene *tbp*. Samples (*n*=3 with 10 embryos per sample) were taken at 1, 2, 3 and 4 hpi of zymosan or PBS as a control. The relative fold change of zymosan- versus PBS-injected samples is shown for each time point to account for a possible wounding effect by the injection itself. (E) qPCR was performed to determine the whole-organism expression level of *il1b*, *tnfa*, *il10* and *tgfb1* relative to the expression of the housekeeping gene *tbp*. Wild-type or *mecp2*-null samples (*n*=3 with 10 embryos per sample) were taken at 1, 2, 3 and 4 hpi of zymosan. The relative fold change of zymosan-injected larvae versus uninjected wild-type controls is shown for each time point to not exclude a potential different response in *mecp2*-null samples towards the wound caused by the injection. One-way ANOVA with Tukey's post hoc test was used for all statistical analyses (***P<0.001; **P<0.01; *P<0.05; ns, not significant; data are representative of at least two individual experiments).

Our finding that *tnfa* is downregulated in *mecp2*-null as early as 6 hpf is highly suggestive of a direct effect of Mecp2 on *tnfa* expression. To test whether the *tnfa* gene has lost its responsiveness to inflammatory stress signals, we designed an acute inflammation assay by injecting the yeast cell wall particle zymosan, a TLR2 ligand (Underhill et al., 1999), into the brains of 3 dpf zebrafish larvae (Fig. 4A). Fluorescently labeled zymosan injected into the brains of wild-type larvae was rapidly phagocytosed by Tg(*mpeg1*:eGFP)-positive microglia (Fig. 4B), and all zymosan particles were cleared from the brain tissue at 4 h postinjection (hpi) (Fig. 4C). The clearance of zymosan is accompanied by an acute inflammatory response characterized by an initial upregulation of the proinflammatory cytokine genes *il1b* and *tnfa*, followed by an upregulation of the anti-inflammatory cytokine genes *il10* and *tgfb1* (Fig. 4D). The gene expression levels of these inflammatory cytokines had returned to baseline levels at 4 hpi of zymosan (Fig. 4D). The inflammatory response to zymosan injected into the brain is strongest in dissected heads of wild-type larvae, but its effects on *il1b* gene expression can also be detected in whole animal preparations of injected zebrafish larvae (Fig. S4A).

We confirmed that Tg(*mpeg1*:eGFP)-positive cells present in the brain of wild-type and *mecp2*-null larvae phagocytosed zymosan at comparable rates (Fig. S4B,C). We then used qPCR to compare the gene expression levels of *il1b*, *il10* and *tnfa* between *mecp2*-null and wild-type larvae over a 4-h time course after injection of zymosan into the brain. Strikingly, *mecp2*-null larvae were unable to increase the gene expression level of *tnfa* in response to zymosan injection, unlike the wild-type control group (Fig. 4E). By comparison, we found no difference in gene expression levels of *il1b* and *il10* between *mecp2*-null and wild-type larvae during this inflammatory response (Fig. 4E). This demonstrates that the genetic regulation of *il1b* and *il10* in response to a danger signal is not disturbed by Mecp2 deficiency, and suggests that their upregulation during *mecp2*-null larval development is part of an inflammatory response to disturbances in tissue homeostasis. We conclude that zebrafish *tnfa* was unresponsive to inflammatory stimulation in the absence of functional Mecp2. These results show that even during an acute stress event, *mecp2*-null larvae cannot activate *tnfa* expression, and further suggests that Mecp2 is required for proper expression of *tnfa*.

### Re-expression of *mecp2* in *mecp2*-null zebrafish embryos partially rescues *tnfa* gene expression

**Fig. 5.**
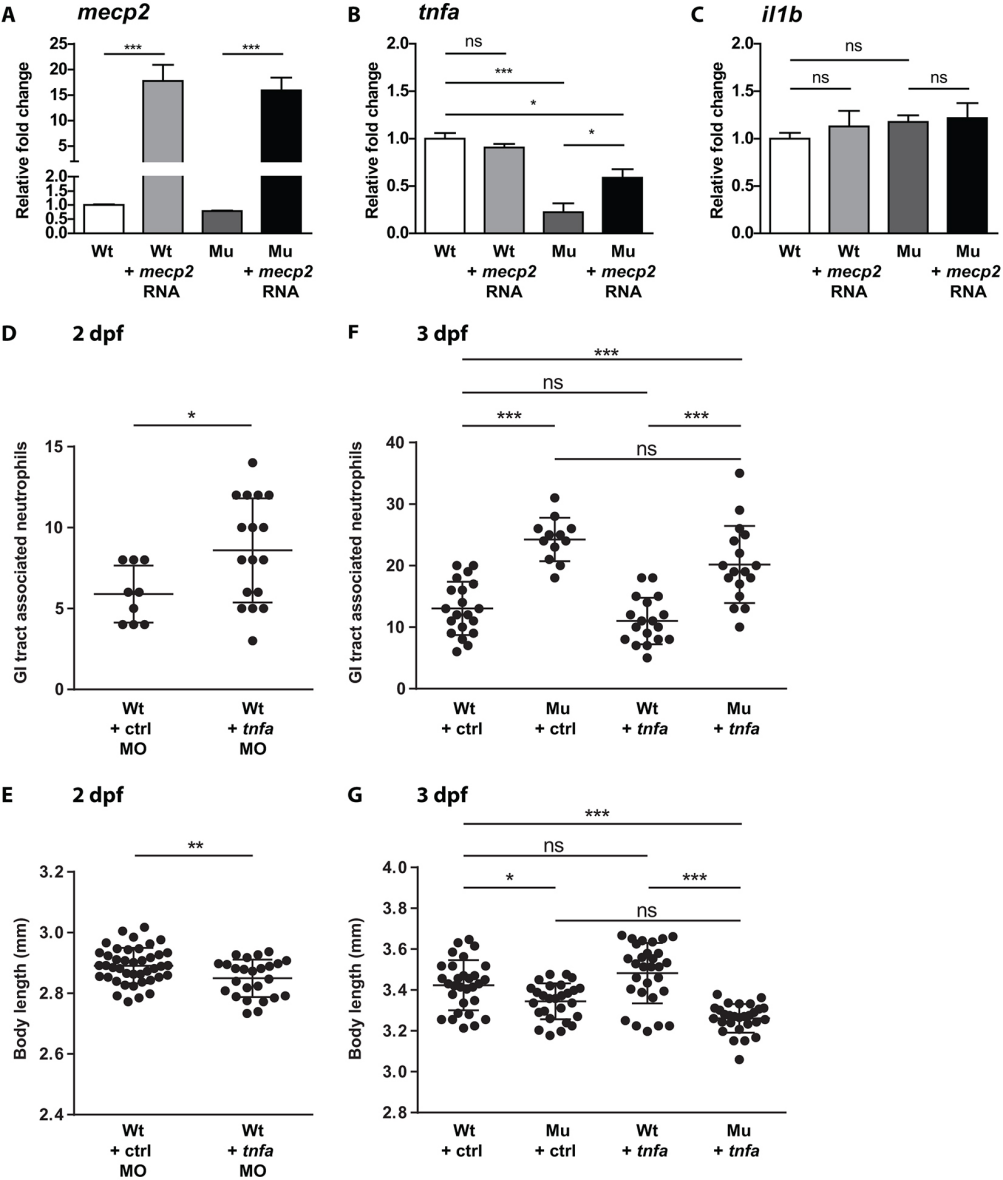
**Re-expression of *mecp2* in *mecp2*-null zebrafish embryos partially rescues *tnfa* gene expression, while enforced expression of *tnfa* does not alleviate phenotypes caused by Mecp2 deficiency.** (A,B,C) Wild-type and *mecp2*-null one-cell stage embryos were injected with 50 pg of full-length *mecp2* mRNA. qPCR was performed to determine the whole-organism gene expression level of *mecp2* (A), *tnfa* (B) and *il1b* (C), relative to the expression of the housekeeping gene *tbp*. Wild-type and *mecp2*-null samples (*n*=3 with 30 embryos pooled per sample) were taken at 24 hpf. The relative fold change of each condition versus uninjected wild-type controls is shown. (D,E) Oligonucleotide morpholino targeting *tnfa* expression was injected as previously described by Candel et al. (2014). Numbers of Tg(*mpx*:eGFP)-positive neutrophils associated with the GI tract of 2 dpf control and *tnfa* morpholino-injected larvae were counted (*n*≥9 embryos per condition) (D), and the total body lengths of 2 dpf control and *tnfa* morpholino-injected larvae (*n*≥25 per condition) were measured (E). (F,G) Wild-type and *mecp2*-null one-cell stage embryos were injected with *tnfa* cDNA-containing plasmids as previously described by López-Muñoz et al. (2011). Numbers of Tg(*mpx*:eGFP)-positive neutrophils associated with the GI tract of 3 dpf wild-type or *mecp2*-null larvae (injected with control or *tnfa* cDNA-containing plasmids) were counted (*n*≥12 embryos per condition) (F), and the total body lengths of the larvae were measured (*n*≥26 embryos per condition) (G). One-way ANOVA with Tukey's post hoc test was used for all statistical analyses involving more than two groups. Student's *t*-test was used for all statistical analyses comparing two groups (***P<0.001; **P<0.01; *P<0.05; ns, not significant; data are representative of at least two individual experiments).

Because *mecp2*-null larvae were unable to express *tnfa* at wild-type levels during development or during an acute inflammatory response, we asked whether re-expression of wild-type *mecp2* was sufficient to restore *tnfa* gene expression levels in *mecp2* mutants. For this purpose, we injected full-length *mecp2* mRNA into *mecp2*-null or wild-type zygotes. Injection of *mecp2* mRNA resulted in a 15- to 20-fold overexpression of *mecp2* at 1 dpf (Fig. 5A), or ∼50-fold overexpression at twice the dose (Fig. S5). Even at the highest dose tested, we were unable to detect increased *mecp2* expression levels at 3 dpf (Fig. S5), suggesting a rapid decay of the injected *mecp2* mRNA. We analyzed the effect of *mecp2* overexpression on *tnfa* and *il1b* gene expression levels by qPCR. We found that overexpression of wild-type *mecp2* in *mecp2*-null could partially rescue *tnfa* gene expression levels at 1 dpf, but did not affect *tnfa* gene expression in wild-type embryos (Fig. 5B). Overexpression of wild-type *mecp2* mRNA had no noticeable effect on gene expression levels of *il1b* in either *mecp2*-null or wild-type embryos (Fig. 5C). The lower *tnfa* expression throughout embryonic and larval development, combined with the unresponsiveness of *tnfa* to inflammatory stimulation in *mecp2*-null larvae, suggested a direct effect of Mecp2 deficiency on *tnfa* gene expression. Based on the *mecp2* mRNA re-expression experiments, we conclude that Mecp2 is required to allow normal expression of *tnfa* in zebrafish embryos and larvae. However, overexpression of *mecp2* mRNA in wild-type embryos did not alter *tnfa* expression, indicating that Mecp2 alone is not sufficient to induce *tnfa* expression. This suggests a mechanism in which Mecp2 allows additional transcriptional regulators to be recruited to modulate *tnfa* gene expression.

### Re-expression of *tnfa* in *mecp2*-null zebrafish embryos does not rescue RTT phenotypes

We observed that *tnfa* expression is significantly reduced in *mecp2*-null zebrafish during embryonic and larval development. The posterior gut epithelium is a prominent source of *tnfa* expression in wild-type larvae (Fig. 3F), whereas *mecp2*-null animals had no detectable *tnfa* expression in this tissue (Fig. 3J). Because dysregulated *tnfa* expression has previously been implicated in the onset of inflammatory bowel disease in zebrafish larvae (Marjoram et al., 2015), we hypothesized that the lack of *tnfa* expression might contribute to the development of inflammatory phenotypes in the GI tract of *mecp2*-null zebrafish. To test for the potential involvement of reduced *tnfa* expression in the development of RTT phenotypes, we injected a previously described morpholino oligonucleotide targeting *tnfa* expression into wild-type zygotes (López-Muñoz et al., 2011). We found that knockdown of *tnfa* resulted in a significant increase in the number of GI tract-associated neutrophils compared to control-injected individuals (Fig. 5D), as well as a significant decrease in total body length (Fig. 5E). Both these phenotypes are also observed in *mecp2*-null larvae. Next, we attempted to rescue the GI tract neutrophil infiltration and growth reduction observed in *mecp2*-null larvae by re-expressing *tnfa*. For this purpose, we injected plasmid encoding full-length *tnfa* mRNA into *mecp2*-null or wild-type zygotes (Fig. S6). Enforced expression of *tnfa* did not reduce the number of neutrophils associated with the GI tract in *mecp2*-null larvae (Fig. 5F), nor did it restore the reduced body length of *mecp2*-null larvae (Fig. 5G). In summary, although knockdown of *tnfa* mimicked the phenotypes observed in *mecp2*-null larvae, restoring *tnfa* expression was not sufficient to rescue the growth retardation and GI tract inflammatory phenotypes observed in *mecp2*-null animals.

### RNA sequencing reveals early developmental effects of *mecp2* deficiency and predicts RTT phenotypes

**Fig. 6.**
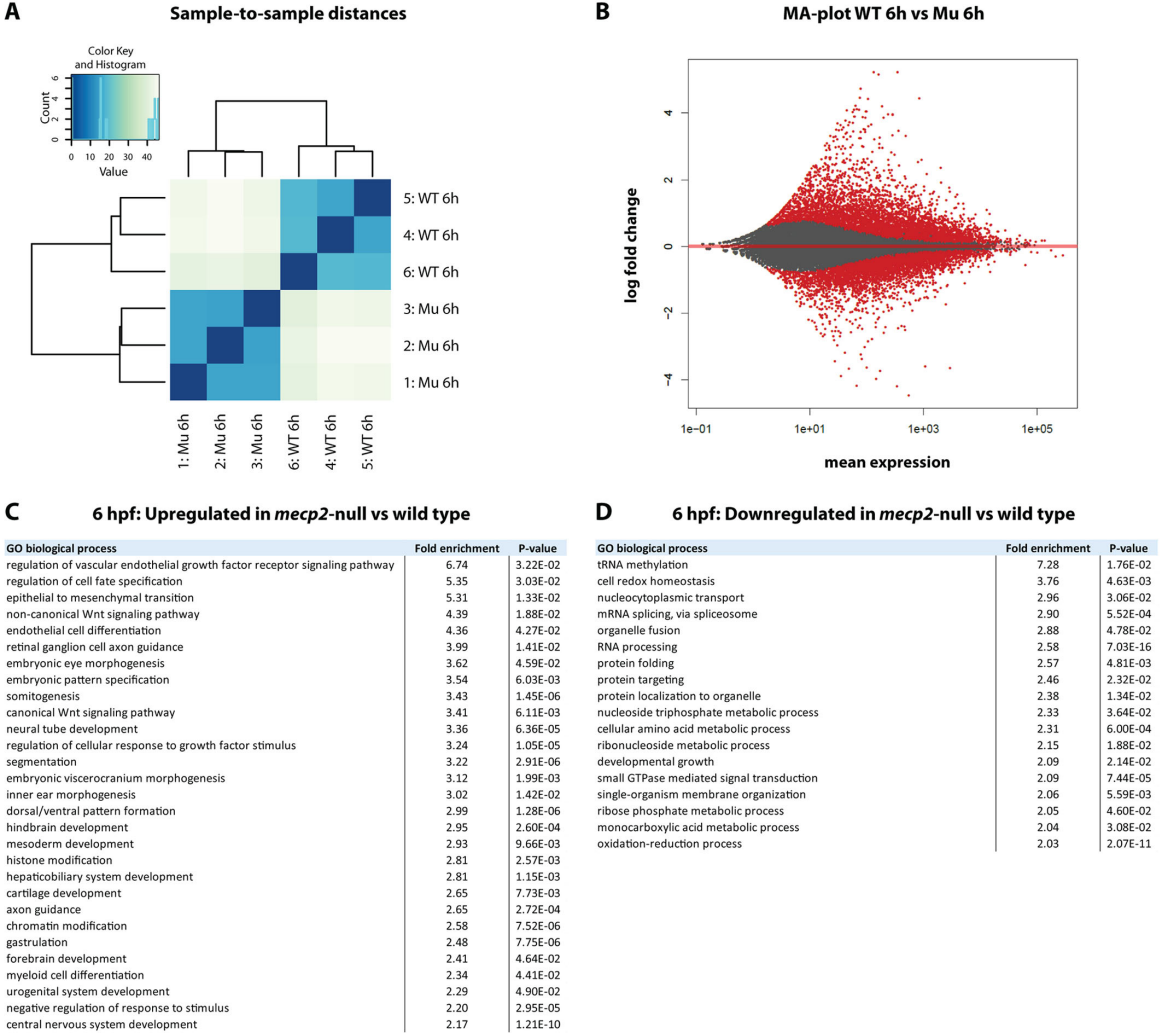
**RNA sequencing reveals early developmental effects of *mecp2* deficiency relevant to RTT.** RNA sequencing was performed on RNA isolated from groups of 6 hpf wild-type and *mecp2*-null embryos (*n*=3 biological replicates per condition with 30 embryos pooled per replicate). DESeq2 analysis was performed using http://usegalaxy.org/. (A) A sample-to-sample distances plot for the three biological replicates per condition was used to detect potential outliers. (B) An MA-plot of differential expression caused by Mecp2 deficiency is shown. The log2 fold change is plotted on the *y*-axis and the average of the counts normalized by size factor is shown on the *x*-axis. Each gene is represented by a dot. Genes with an adjusted *P* value <0.05 are shown in red. (C,D) Enriched GO processes for significantly up- or downregulated genes in *mecp2*-null versus wild-type embryos are listed in the tables. Only GO terms with at least twofold enrichment are shown. For hierarchically clustered GO terms, only the most specific term is included in the list.

In this study, we have demonstrated that Mecp2 regulates *tnfa* gene expression levels during early zebrafish embryonic development. Even at 6 hpf, when low levels of *tnfa* expression can first be detected in wild-type embryos, *mecp2*-null embryos express significantly reduced levels of *tnfa*. At this time point of development, zebrafish embryos are undergoing epiboly and gastrulation, which initiate formation of the basic body plan. Although we were able to detect the effect of *mecp2* deficiency at this early developmental stage in zebrafish embryos, RTT patients do not display phenotypes until at least 6 months after birth. Therefore, we reasoned that the zebrafish embryo could be highly informative regarding the earliest effects of disrupted MECP2-function that ultimately result in disease phenotypes. To assess the genes that are disrupted during early development of *mecp2*-null, we performed RNA sequencing to reveal whole-transcriptome differences between 6 hpf *mecp2*-null and wild-type embryos. For the *mecp2*-null group, embryos were derived from homozygous *mecp2*-null parents to avoid the confounding influence of maternally deposited wild-type *mecp2* RNA. The three biological replicates of each condition clustered closely together after DESEq2 analysis (Fig. 6A). At 6 hpf, 3837 transcripts were significantly upregulated in *mecp2*-null versus wild-type embryos, whereas 4217 transcripts were significantly downregulated (Fig. 6B). Although the raw counts for *tnfa* were lower in *mecp2*-null compared to wild-type embryos, the average number of raw counts for *tnfa* in wild-type embryos was too low to demonstrate significance (data not shown).

For an unbiased assessment of potentially disrupted biological processes in *mecp2*-null embryos at 6 hpf, we submitted the subsets of differentially up- or downregulated genes to gene ontology (GO) analysis. GO analysis revealed that genes associated with a large range of biological processes were significantly enriched in the differentially expressed subsets, illustrating that *mecp2* deficiency has a broad effect on transcription. We limited our further analysis to GO terms with at least twofold enrichment, and only included the most specific GO term for groups of hierarchically clustered terms (Fig. 6C,D). This strict GO term analysis revealed significantly enriched biological processes that are linked to known MECP2-functions, such as epigenetic regulation of transcription and mRNA splicing (Lyst and Bird, 2015). Importantly, GO analysis also identified enriched processes at this early developmental stage, which become relevant to RTT phenotypes at later stages, including neurological development, craniofacial development, vascular dysfunction, redox homeostasis, developmental growth, myeloid cell differentiation and hepatobiliary system development. Finally, GO analysis identified enriched biological processes that, to the best of our knowledge, have not been previously linked to MECP2-function or RTT, including dorsal/ventral pattern formation; protein folding; and intracellular protein targeting. The analysis of the RNA sequencing data underscores the relevance of the zebrafish model for the study of MECP2 function and RTT, while potentially identifying new biological processes of interest.

**Fig. 7.**
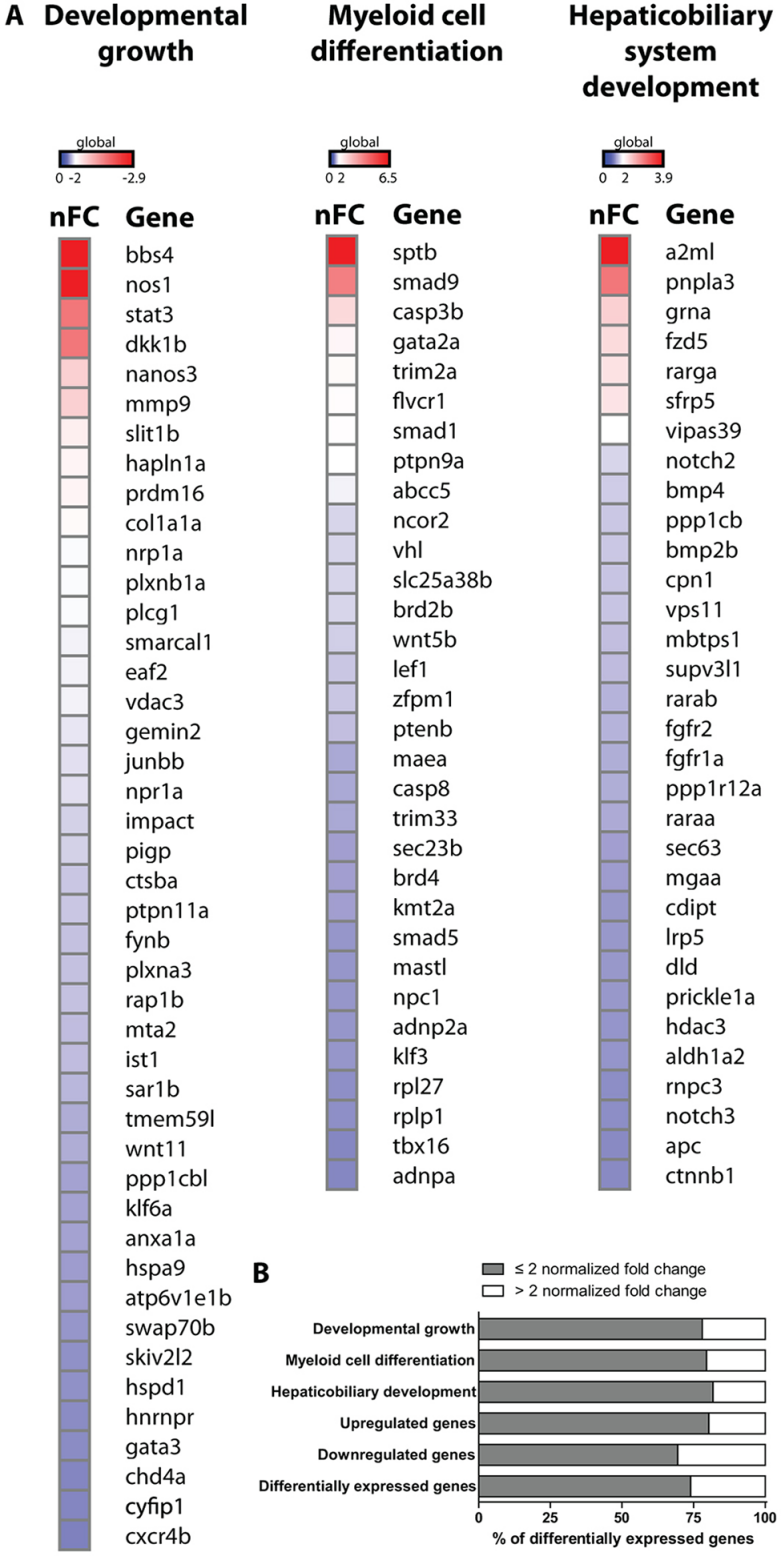
**Heatmap of differentially expressed genes in *mecp2*-null embryos.** (A) Heatmap displaying the extent of differential gene expression between 6 hpf *mecp2*-null versus wild-type embryos. The genes incorporated in the heatmap represent all differentially expressed genes that belong to the GO terms ‘developmental growth’ (downregulated genes), ‘myeloid cell differentiation’ (upregulated genes) and ‘hepaticobiliary system development’ (upregulated genes). For all genes, the positive or negative normalized fold change (nFC) for *mecp2*-null embryos versus wild-type embryos is shown. (B) The graph displays the percentage of significantly differentially expressed genes in *mecp2*-null versus wild-type embryos with a fold change ≤2 or >2. The following groups are shown: significantly differentially expressed genes belonging to the GO terms ‘developmental growth’, ‘myeloid cell differentiation’ and ‘hepaticobiliary system development’; genome-wide significantly upregulated genes; genome-wide significantly downregulated genes; and all significantly differentially expressed genes.

At the same time, the unbiased analysis of enriched GO terms appears predictive for the growth retardation, myeloid cell number disruption (neutrophilia) and hepatobiliary dysfunction that occurs later during development. For the GO term ‘developmental growth’, 44 out of a total of 187 genes linked to this biological process were significantly downregulated in 6 hpf *mecp2*-null versus wild-type embryos. For the GO terms ‘myeloid cell differentiation’ and ‘hepaticobiliary system development’, 32 out of a total of 133 or 111 genes linked to this process, respectively, were significantly upregulated in *mecp2*-null embryos. To investigate the extent of individual gene dysregulation in the absence of Mecp2, we plotted the normalized fold change in all differentially expressed genes linked to these three GO terms in a heatmap (Fig. 7A). Mecp2 deficiency affected the expression levels of a large number of genes related to these biological processes; ∼75% of these genes were up- or downregulated, at a twofold change or less (Fig. 7B). The same observation was made on a genome-wide scale for all significantly upregulated genes in *mecp2*-null embryos, or to a lesser degree for all significantly downregulated genes (Fig. 7B). Notable exceptions to this general tendency for small differences in gene expression levels are *bbs4* and *nos1* (GO term ‘developmental growth’); *sptb*, *smad9* and *casp3b* (GO term ‘myeloid cell differentiation’); and *sfrp5* and *a2ml* (GO term ‘hepaticobiliary system development’). While the neuron-expressed Nitric oxide synthase 1 (Nos1) protein is well known for its role in neurotransmission, mutations in genes from the Bardet-Biedl syndrome (BBS) family, such as *bbs4*, result in an autosomal recessive disorder characterized by mental retardation and other severe symptoms. The anti-inflammatory adipocytokine Sfrp5 modulates metabolic dysfunction during obesity in mice (Ouchi et al., 2010), and *a2ml* (Alpha2 macroglobulin-like) was shown to be essential for liver development in zebrafish (Hong and Dawid, 2008). A potential role for Sptb, Smad9 and Casp3b in neutrophilia is not directly clear. For all genes with relatively high differential expression between *mecp2*-null and wild-type embryos at 6 hpf, it will be interesting to investigate whether their dysregulation extends into developmental phases when RTT phenotypes first arise.

## DISCUSSION

The large body of literature on MECP2 and RTT contains evidence that mutations in MECP2, as well as its overexpression caused by duplication of its genetic locus, result in abnormal functioning of the immune system (Bauer et al., 2015; Cortelazzo et al., 2014; Cronk et al., 2015; Derecki et al., 2012; Leoncini et al., 2015; Pecorelli et al., 2016). Furthermore, because RTT patients acquire disease symptoms after an apparently normal early development, we hypothesized that misregulated responses to external or internal inflammatory stimuli encountered during development could play a key role in the onset of RTT. We therefore set out to test the potential function of zebrafish Mecp2 as an epigenetic regulator of immune and inflammatory responses during development.

Indeed, gene expression levels of the inflammation marker *crp* and mobilization of neutrophils provided evidence for the presence of inflammation in *mecp2*-null larvae after an inflammation-free early development. Increased gene expression levels of *il1b* and *il10* measured at a whole-organism level were found to be involved in this inflammatory response. We hypothesized that, when lacking the epigenetic regulator Mecp2, the zebrafish genes encoding Il1b and Il10 were hyper-responsive to inflammatory stimulation. By submitting both wild-type and *mecp2*-null larvae to an acute inflammation assay, we were able to disprove this hypothesis. The expression levels of *il1b* and *il10* were regulated in a similar fashion in response to inflammatory stimulation in wild-type and *mecp2*-null larvae. Combined with the fact that *il1b* and *il10* were expressed at wild-type levels in *mecp2*-null embryos during early development, we suggest that the peak in expression of these pro- and anti-inflammatory cytokines was a response to a disturbance in tissue homeostasis in the absence of Mecp2.

We observed an increased infiltration of neutrophils into the GI tract of *mecp2*-null larvae, combined with GI tract phenotypes and a potential disturbance of bile production or flow. These observations are relevant, because RTT patients frequently display GI tract phenotypes, including GI dismotility (Baikie et al., 2014). Additionally, cholesterol metabolism is altered in RTT patients (Segatto et al., 2014), and limiting cholesterol biosynthesis alleviated RTT symptoms and increased the survival of *mecp2*-null mice (Buchovecky et al., 2013). While bile acids, a major component of cholesterol, have immunomodulatory effects (Brestoff and Artis, 2013), inflammation can also suppress the expression of bile transporters and thereby reduce the flow of bile (Kosters and Karpen, 2010). With the proven contribution of zebrafish larval and embryonic models to the study of liver diseases and inflammatory bowel diseases (Goessling and Sadler, 2015; Love et al., 2007), the zebrafish *mecp2*-null mutant might be ideally suited to illuminating the role of inflammation in the GI tract of RTT patients.

The most striking result obtained during this study was that zebrafish *tnfa* was not expressed at normal levels in the absence of functional Mecp2 during embryonic and larval development, or during an acute inflammatory response. Combined with our finding that re-expression of wild-type Mecp2 can partially rescue *tnfa* expression in *mecp2*-null embryos, we conclude that zebrafish Mecp2 influences the transcriptional potential of *tnfa*. Importantly, the dysregulated expression levels of *tnfa* at 6 hpf precede any of the developmental phenotypes observed in the absence of functional Mecp2, and could potentially be a causative factor for RTT features displayed later during development. Indeed, knockdown of *tnfa* gene expression induced neutrophilic infiltration into the GI tract of zebrafish larvae, a phenotype resembling that observed in *mecp2*-null individuals. In this light, it is interesting to note that genetic inhibition of Tnfa and Tnfr2 in zebrafish previously resulted in the mobilization of neutrophils to the skin, revealing a crucial role for the TNFα/TNFR2 axis in the protection against Duox1-mediated oxidative stress (Candel et al., 2014). RTT patients often display oxidative stress, and we identified the GO term ‘redox homeostasis’ as one of the biological pathways altered in *mecp2*-null embryos. The potential link between reduced *tnfa* expression in the GI tract and inflammation caused by increased oxidative stress is therefore an interesting topic for further study in *mecp2*-null zebrafish embryos and larvae.

However, we found that re-expression of *tnfa* did not alleviate the phenotypes observed in *mecp2*-null zebrafish. Transcriptome analysis revealed that a total of 8054 genes are differentially expressed between *mecp2*-null embryos and wild types at 6 hpf. Even if the enforced expression of *tnfa* could be titrated to match wild-type endogenous levels, which differ according to tissue and circumstance, it indeed seems unlikely that re-expression of only one dysregulated gene would be sufficient to alleviate the observed RTT features.

The observation that overexpression of *mecp2* in wild-type embryos did not raise *tnfa* gene expression levels indicates that the presence of Mecp2 alone is not sufficient to increase transcription of *tnfa*. The experiments performed in this study also provide clues into which aspect of the diverse Mecp2 functions might be involved in the regulation of *tnfa* (Lyst and Bird, 2015). The Tg(*tnfa*:eGFP) construct (Marjoram et al., 2015), introducing an additional copy of the *tnfa* promoter in the genome, did not drive expression of eGFP in the absence of Mecp2, indicating that the regulatory sequences of the *tnfa* transgene are critically important for its regulation by Mecp2. It is possible that sequence-specific DNA-binding of Mecp2 results in chromatin remodeling that increases the transcriptional potential of the zebrafish *tnfa* gene (Ballestar et al., 2000; Baubec et al., 2013; Yusufzai and Wolffe, 2000). Another plausible explanation is that Mecp2 is involved in the transcriptional activation of *tnfa* by recruiting the co-activator CREB1, since the CREB-binding protein (CBP)/p300 was shown to play a stimulus-dependent role in T cell receptor-activated TNFα gene expression (Falvo et al., 2000).

Several *in vivo* and *in vitro* models exist for the study of RTT and MECP2 function, including *Mecp2*-null mutant mice (Chen et al., 2001; Guy et al., 2001); *Xenopus laevis* with truncated Mecp2 (Stancheva et al., 2003); induced pluripotent stem cells (iPSCs) from RTT patients' fibroblasts (Marchetto et al., 2010); *mecp2*-null mutant zebrafish (Pietri et al., 2013); and most recently transgenic monkeys overexpressing MECP2 (Liu et al., 2016). The results obtained using these different models are sometimes conflicting and Mecp2 function varies between different tissues or cells of the same organism. For instance, the NFκB-pathway component *Irak1* was specifically upregulated in cortical callosal projection neurons in *Mecp2*-null mice, but not in distinct organs such as the lungs, heart, spleen or kidney (Kishi et al., 2016). Even when the same model organism and experimental conditions are used, results can still differ fundamentally (Derecki et al., 2012; Wang et al., 2015). In this regard, while we consistently found zebrafish *tnfa* to be downregulated in *mecp2*-null animals, Cronk et al. (2015) found an increase in Tnfa-induced transcriptional signature genes specifically in isolated *Mecp2*-null microglia. The different cell source utilized in these experiments might explain the conflicting results, making it worthwhile to analyze *tnfa* transcript levels in isolated zebrafish *mecp2*-null microglia and other immune cells.

With the sometimes conflicting findings on the effect of Mecp2 deficiency under differing conditions and from various model systems, it is challenging to reach a unified and evolutionary conserved conclusion on Mecp2 function. Nonetheless, we believe that contributions from each individual model system will ultimately help to understand the function of MECP2 in health and disease. We have used the zebrafish embryonic and larval system to demonstrate that Mecp2 is required for *tnfa* expression during zebrafish development and inflammation. Besides this, our RNA sequencing results provide insights into the earliest genetic alterations that occur in the absence of MECP2 function, which ultimately could result in RTT phenotypes. Furthermore, zebrafish embryos are amenable to high-throughput screening for drugs with the potential to remedy these phenotypes (Tan and Zon, 2011). We believe that these findings have the potential to instruct future studies in zebrafish and other model systems to increase our understanding of MECP2 function and its role in RTT pathogenesis.

## MATERIALS AND METHODS

### Zebrafish husbandry and maintenance

Zebrafish (*Danio rerio*) were maintained and used for experiments according to the guidelines of the UCSD Institutional Animal Care and Use Committee. The following zebrafish lines were used: AB (wild-type strain); mecp2^Q63*^ mutants (Pietri et al., 2013); Tg(*mpx*:eGFP)*^i114^* (Renshaw et al., 2006); Tg(*mpeg1*:eGFP)^gl22^ (Ellett et al., 2011); Tg(*tnfa*:eGFP) (Marjoram et al., 2015). Genotyping of mecp2^Q63*^ mutants occurred as previously described (Pietri et al., 2013). When needed for experimental purposes, zebrafish were anesthetized using Tricaine (200 µg/ml).

### Microscopy

For stereomicroscopy, embryos and larvae were mounted in E3 medium containing 3% methyl cellulose (Sigma-Aldrich). Brightfield images were acquired using a Leica MZ16 stereomicroscope with a Leica DFC295 camera (Leica Microsystems). Epifluorescence images were acquired using an AxioZoom.V16 stereomicroscope (Zeiss). For confocal microscopy, larvae were mounted in E3 medium containing 0.5% low melting point agarose (Sigma-Aldrich). Confocal micrographs were acquired using a Leica SP5 confocal system (Leica Microsystems). Images were created using Imaris (Bitplane) and ImageJ (https://imagej.nih.gov/ij/) software.

### qPCR

mRNA was isolated using the RNeasy mini kit according to the manufacturer's instructions (Qiagen). cDNA was synthesized using the iScript cDNA synthesis kit according to the manufacturer's instructions (BioRad). qPCR was performed using iQ SYBR Green Supermix (BioRad) and the BioRad CFX96 real-time system according to the manufacturer's instructions. Gene expression levels were calculated relative to the expression of the housekeeping gene *TATA box* *binding protein* according to the 2^−ΔΔCt^ method. Primers used for qPCR analysis of gene expression are listed in Table S1.

### Microinjection of zymosan particles

Zebrafish larvae of 3 dpf were positioned with the dorsal side up to allow injection of 1 nl PBS containing 100-150 Alexa Fluor 594-labeled Zymosan A (*S. cerevisiae*) BioParticles (Molecular Probes) into the brain. As a control for a potential wounding effect, 1 nl of sterile PBS was injected in a similar manner. The percentage of zymosan particles phagocytosed by Tg(*mpeg1*:eGFP)-positive cells was determined based on confocal micrographs of the brain.

### Microinjection of mRNA, plasmids and antisense oligonucleotide morpholinos

A gBlock (Integrated DNA Technology) containing full-length zebrafish *mecp2* cDNA (ENSDART00000040672) was cloned into a Zero Blunt TOPO PCR vector according to the manufacturer's instructions (Life Sciences). Zebrafish *mecp2* mRNA was synthesized using the mMessage mMachine SP6 Transcription Kit according to the manufacturer's instructions (Invitrogen). Then, 50 pg or 100 pg *mecp2* mRNA was injected into the yolk of one-cell stage zebrafish embryos. The antisense oligonucleotide morpholino targeting *mecp2* expression was injected as described by Gao et al. (2015), while the antisense oligonucleotide morpholino targeting *tnfa* expression was injected as described by López-Muñoz et al. (2011). Control plasmid (pCS2+) and Tnfa plasmid (Roca et al., 2008) (20 pg) were injected into the yolk sac of one-cell stage embryos.

### RNA sequencing

mRNA was isolated using the RNeasy mini kit according to the manufacturer's instructions (Qiagen). Library preparation and sequencing was performed by the Institute for Genomic Medicine Center at the University of California, San Diego. RNA sequencing was performed on an Illumina HiSeq4000 platform using single reads of 50 bases in length. RNA sequencing data were mapped to the zebrafish genome (version Zv9) using TopHat 2.1.1 (http://ccb.jhu.edu/software/tophat/index.shtml). Raw counts were submitted to DESeq2 analysis using the Galaxy website (http://usegalaxy.org/). GO analysis was performed using the Gene Ontology website (http://geneontology.org/). The heatmap displaying differential gene expression was created using Gene-E software (Broad Institute; https://software.broadinstitute.org/GENE-E/). RNA sequencing data are accessible under Gene Expression Omnibus accession number GSE80348.

### Statistical analysis

All data (mean±s.e.m.), except for the RNA sequencing data, were analyzed (Prism 5.0, GraphPad Software) using unpaired, two-tailed Student's *t*-tests for comparisons between two groups, or one-way ANOVA with Tukey's Multiple Comparison method as a post hoc test for other data (***P<0.001; **P<0.01; *P<0.05; ns, not significant).

## Supplementary Material

Supplementary information
