# Supplementary material for: Mecp2 regulates tnfa during zebrafish embryonic development and acute inflammation
Source: Dis Model Mech. 2017 Dec 1;10(12):1439–51. doi: 10.1242/dmm.026922 (PMC5769600; doi:10.1242/dmm.026922)
Supplement: Supplementary information [file dmm-10-026922-s1.pdf]

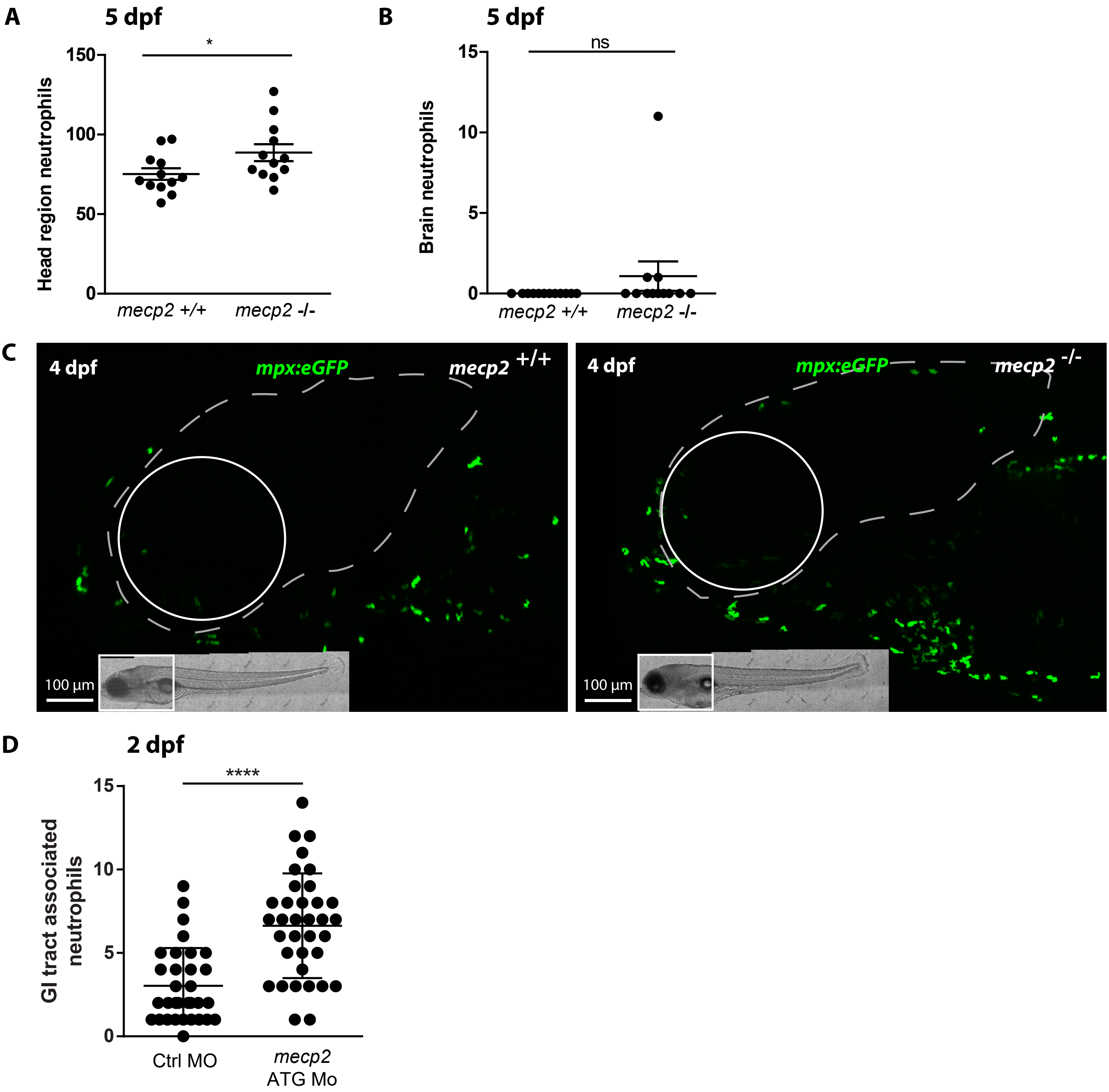

Supplementary figure 1: Neutrophil numbers in the head and brain region of wild type and *mecp2*-null larvae

(A) Tg(*mpx*:eGFP)-positive neutrophils were enumerated in the head region of 5 dpf wild type and *mecp2*-null larvae using stereo fluorescent microscopy (n=12 embryos per condition). **(B)** Numbers of Tg(*mpx*:eGFP)-positive neutrophils present in the brain were counted for wild type and *mecp2*-null larvae at 5 dpf (n=12 embryos per condition). **(C)** Representative stereo microscopy images of the head region of 5 dpf Tg(*mpx*:eGFP) wild type and *mecp2*-null larvae in which the brain tissue has been indicated with a white dotted line. A Mann–Whitney U test was used for statistical analysis (*: $p<0.05$; ns: not significant). **(D)** Oligonucleotide morpholino targeting *mecp2* expression was injected as previously described by Gao et al. (2015). Numbers of Tg(*mpx*:eGFP)-positive neutrophils associated with the gastrointestinal tract of 2 dpf control and *mecp2* morpholino injected larvae were counted (n≥30 embryos per condition). Student T-test was used for the statistical analyses (***: $p<0.001$).

Disease Models & Mechanisms • Supplementary information

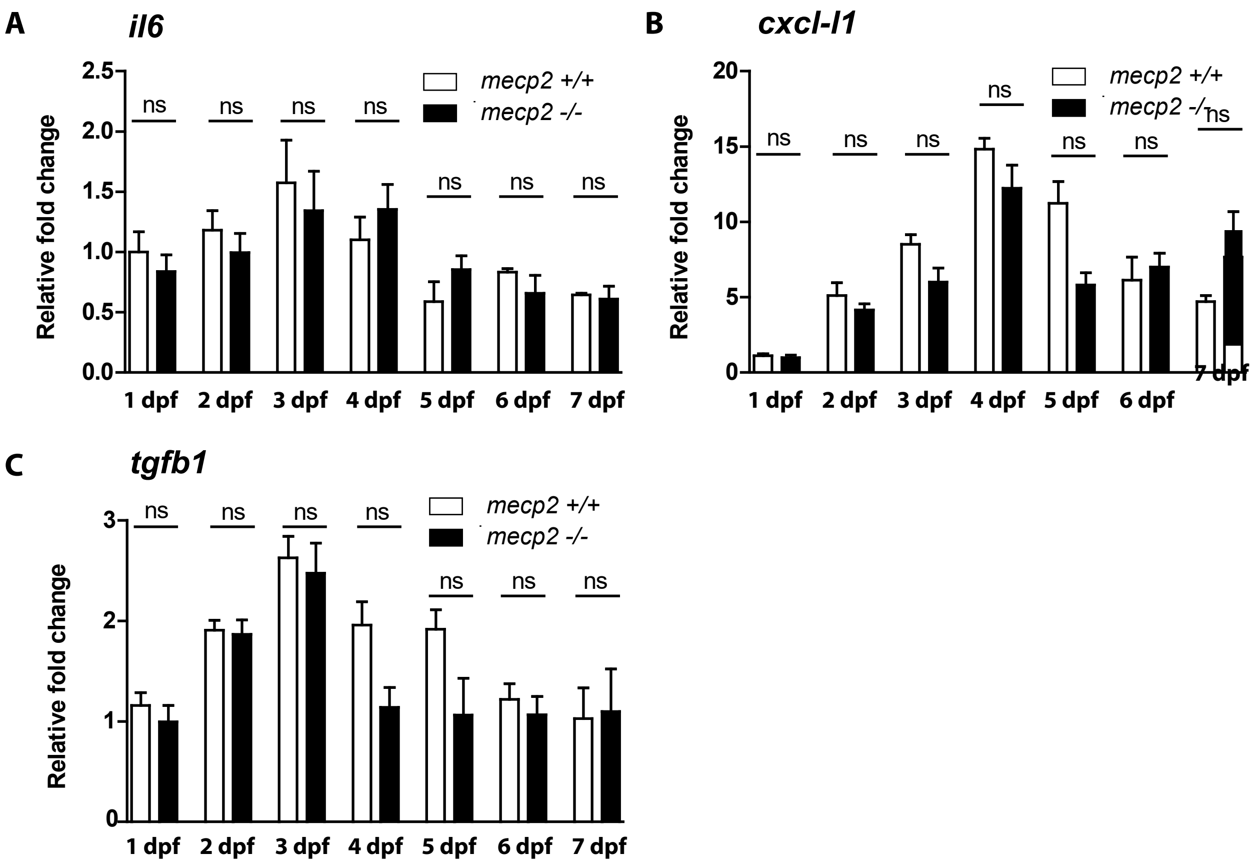

Supplementary figure 2: Expression levels of *il6*, *cxcl8a*, *tgfb1*

Quantitative real-time PCR was performed to determine the whole-organism gene expression level of **(A)** *il6,* **(B)** *cxcl8a*, and **(C)** *tgfb1* in wild type or *mecp2*-null animals. Gene expression is related to the expression of the housekeeping gene *tbp*, while the fold change relative to gene expression in 1 dpf wild type embryos is shown (n=3 with 20 embryos or larvae pooled per sample). A One-way ANOVA with Tukey's post hoc test was used for all statistical analyses (ns: not significant).

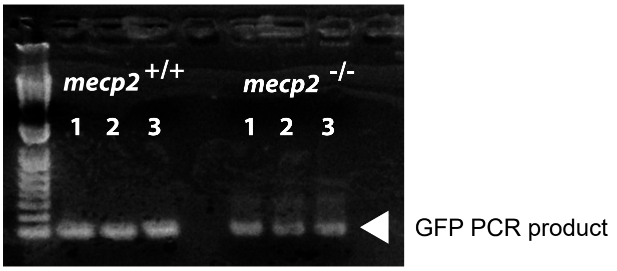

Supplementary figure 3: Confirming the presence of Tg(*tnfa*:eGFP) in *mecp2*-null larvae

Since *mecp2*-null larvae display reduced *tnfa* gene expression levels and reduced Tg(*tnfa*:eGFP) encoded GFP expression, the presence of the Tg(*tnfa*:eGFP) construct in *mecp2*-null larvae was confirmed by genotyping with the following primers: ACGACGGCAACTACAAGACC (forward) and GTCCTCCTTGAAGTCGATGC (reverse). The resulting PCR product was detected using gel electrophoresis. The *mecp2*-null larvae were positive for the Tg(*tnfa*:eGFP) transgene, which corroborates the residual GFP expression observed in mecp2-null larvae with confocal microscopy (Figure 3).

Disease Models & Mechanisms • Supplementary information

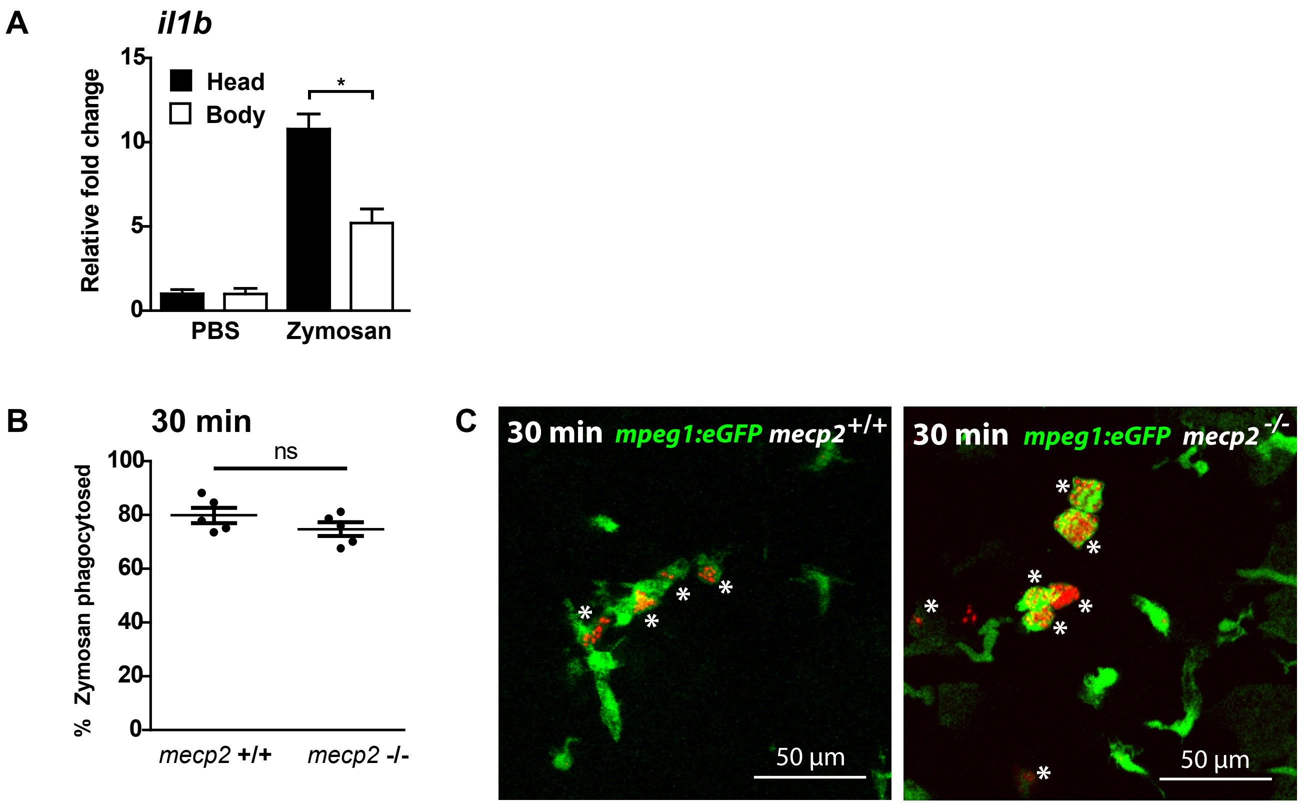

Supplementary figure 4: Microinjection of zymosan into the brain of zebrafish larvae

(A) Zymosan was injected into the brain of 3 dpf wild type larvae. Quantitative real-time PCR was performed to determine gene expression level of *il1b* relative to the expression of the housekeeping gene *tbp* in the dissected heads and bodies of injected larvae. Samples (n=3 with 10 heads or bodies per sample) were taken at 1 hour post injection of zymosan or PBS as a control. The relative fold change of zymosan versus PBS injected samples is shown to account for a possible wounding effect by the injection itself. A One-way ANOVA with Tukey's post hoc test was used for statistical analysis (*: p<0.05; ns: not significant). **(B)** The percentage of zymosan particles phagocytes by Tg(*mpeg1*:eGFP)-positive cells for wild type and *mecp2*-null larvae using confocal microscopy of samples fixed 30 minutes after injection (n=5 larvae per condition). A Student T-test was used for statistical analysis (ns: not significant). **(C)** Representative confocal micrographs of a wild type and *mecp2*-null Tg(*mpeg1*:eGFP) larvae at 30 minutes post injection. An asterix (*) indicates zymosan phagocytosed by Tg(*mpeg1*:eGFP)-positive cells.

Disease Models & Mechanisms • Supplementary information

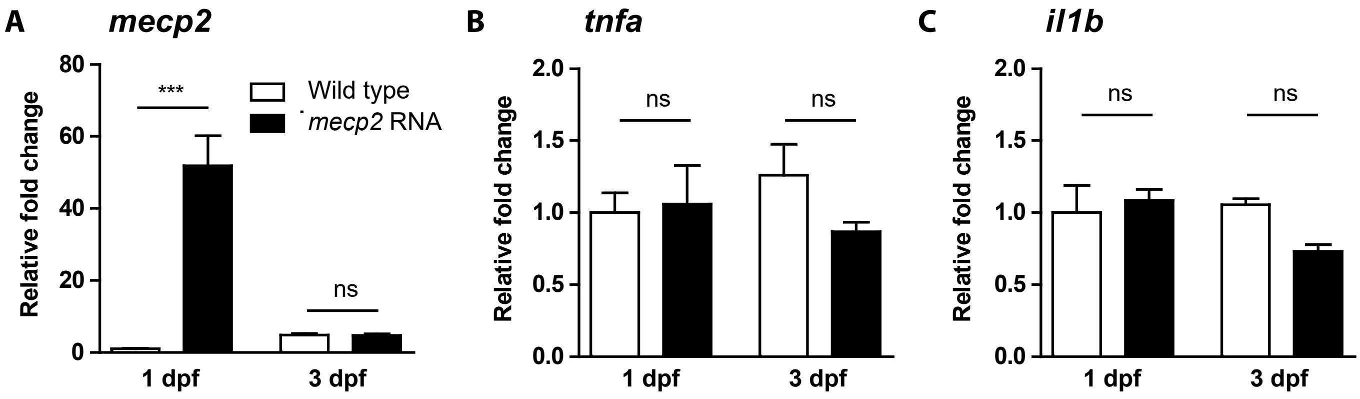

Supplementary figure 5: over-expression of *mecp2* in wild type zebrafish embryos

Wild type 1 cell stage embryos were injected with 100 pg of full length *mecp2* mRNA. Quantitative real-time PCR was performed to determine the whole-organism gene expression level of **(A)** *mecp2*, **(B)** *tnfa*, and **(C)** *il1b* relative to the expression of the housekeeping gene *tbp*. Wild type and RNA-injected samples (n=3 with 30 embryos pooled per sample) were taken at 1 and 3 days post fertilization. The relative fold change of each condition versus uninjected wild type controls is shown. A One-way ANOVA with Tukey's post hoc test was used for all statistical analyses (***: p<0.001; ns: not significant).

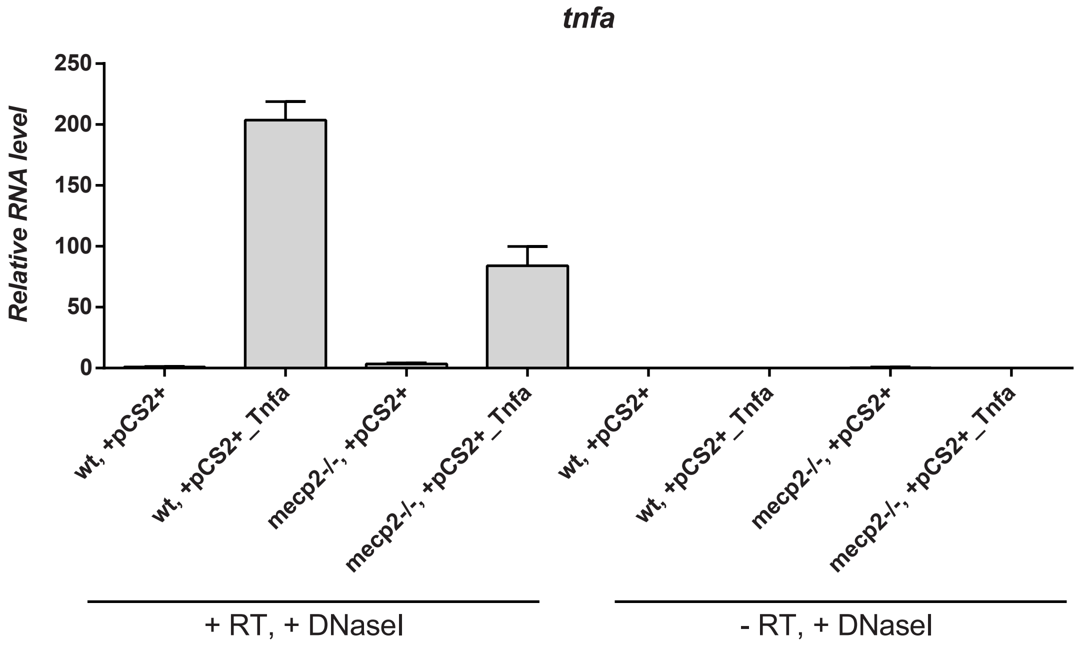

Supplementary figure 6: over-expression of *tnfa* in *mecp2*-null zebrafish embryos

Wild type (wt) and *mecp2*-null (mecp2-/-) 1-cell stage embryos were injected with 20 pg of plasmid containing full length *mecp2* cDNA (+pCS2+_Tnfa), or an empty plasmid as control (+pCS2+). Plasmid-injected samples (n=10 embryos pooled per sample) were taken at 2 days post fertilization. RNA was isolated from these samples and DNaseI treatment was performed to prevent carryover of plasmid DNA (+ DNaseI). Quantitative real-time PCR was performed to determine the whole-organism gene expression level of *tnfa* relative to the expression of the housekeeping gene *ef1a*. Non-reverse transcriptase treated samples (- RT) were taken along as a control to exclude plasmid DNA contamination.

Supplementary table 1: Primers used for quantitative real-time PCR

Gene	Accession number	Forward primer	Reverse primer
crp	NM_001045860.1	GGGTGGACGGTCAACGCAGT	ACGGTGCCGCCAGGACGAAT
cxcl8a	XM_001342570.5	GCTGGATCACACTGCAGAAA	TGCTGCAAACTTTTCCTTGA
il1b	NM_212844.2	GAACAGAATGAAGCACATCAAACC	ACGGCACTGAATCCACCAC
il6	NM_001261449.1	TCAACTTCTCCAGCGTGATG	TCTTTCCCTCTTTTCCTCCTG
il10	NM_001020785.2	ATTTGTGGAGGGCTTTCCTT	AGAGCTGTTGGCAGAATGGT
mecp2	NM_212736.1	ACGTCTACCTTATCAACCCAGA	CCTTCCACGTCCAGAGGG
tbp	NM_200096.1	TCACCCCTATGACGCCTATC	CAAGTTGCACCCCAAGTTT
tgfb1	XM_687246.6	TTTCGGAAAGATCTGGGTTG	AAAGAATTGGCAGAGGGTCA
tnfa	NM_212859.2	GCGCTTTTCTGAATCCTACG	TGCCCAGTCTGTCTCCTTCT
